# Exploring the Role of Acute Exercise-Induced Myokine Release in Glucose Metabolism and Insulin Sensitivity in Healthy and Diabetic Individuals

**DOI:** 10.7759/cureus.78991

**Published:** 2025-02-14

**Authors:** Vaishali Garg, Richa Ghay, Gurdev Goyal, Reena V Saini

**Affiliations:** 1 Physiology, Sri Guru Ram Das Institute of Medical Sciences and Research, Amritsar, IND; 2 Physiology and Medical Education, Sri Guru Ram Das Institute of Medical Sciences and Research, Amritsar, IND; 3 Physiology, Maharishi Markandeshwar (Deemed to be University), Ambala, IND; 4 Biosciences and Technology, Maharishi Markandeshwar (Deemed to be University), Ambala, IND

**Keywords:** exercise, fractalkine, interleukin 6, myokines, type 2 diabetes

## Abstract

Background

Exercise plays a significant role in influencing muscle metabolism and the secretion of myokines, which may have important therapeutic implications for managing type 2 diabetes mellitus (T2DM). This study explores the effects of a single session of moderate-intensity exercise on the levels of circulating myokines, specifically interleukin-6 (IL-6) and fractalkine, in individuals with T2DM compared to healthy controls.

Methodology

A total of 70 participants, including 35 individuals (50%) with T2DM and 35 healthy controls (50%) were enrolled in the study after taking their written informed consent. They took part in a 30-minute treadmill exercise session. Blood samples were collected before and after the exercise to measure fasting blood sugar (FBS), insulin, Homeostatic Model Assessment of Insulin Resistance (HOMA-IR), IL-6, and fractalkine levels, allowing for an assessment of the exercise's effects on both groups.

Results

In the post-exercise period, both groups demonstrated significant improvements in FBS, insulin levels, and HOMA-IR. Notably, IL-6 levels increased, while fractalkine levels decreased, indicating exercise's beneficial metabolic and anti-inflammatory effects. However, it is important to note that no significant correlation was observed between myokine levels and the markers of glucose metabolism.

Conclusions

This study demonstrates that acute exercise positively impacts glucose regulation and myokine modulation in both T2DM and healthy individuals. The findings support the inclusion of exercise as an effective strategy for improving metabolic health in diabetes management, highlighting the role of muscle-derived myokines in regulating glucose metabolism. Further research is needed to explore the long-term effects and mechanistic pathways involved.

## Introduction

Type 2 diabetes mellitus (T2DM) is a global health crisis affecting over 400 million people, with projections suggesting a rise to 700 million by 2045 [1]. The disease ranks among the top 10 causes of death and disability worldwide [2]. Characterized by hyperglycemia, T2DM leads to a range of metabolic dysfunctions and heightened inflammation, contributing to severe complications. Lifestyle shifts significantly drive the escalating prevalence of T2DM, particularly reduced physical activity levels leading to skeletal muscle insulin resistance [3].

Regular physical activity, particularly exercise, plays a critical role in managing T2DM [4]. During exercise, skeletal muscles, which constitute a significant portion of body mass, serve as the primary sites for glucose uptake. This process enhances insulin sensitivity, reduces metabolic disturbances, and optimizes the functioning of the endocrine pancreas [5]. This process occurs through insulin-independent mechanisms, improving overall glycemic control. Glycemic control is crucial for averting long-term complications, reducing premature mortality, and enhancing overall health in individuals with diabetes [6,7].

The role of physical activity in T2DM management is well-documented. Health authorities like the American Diabetes Association (ADA) and the World Health Organization (WHO) advocate for at least 150-300 minutes of moderate-intensity aerobic activity weekly, complemented by strength training sessions. Brisk walking, for example, is frequently recommended for patients with T2DM as a moderate-intensity exercise [2,8].

The physiological benefits of exercise are partly mediated through the secretion of myokines, cytokines, and other peptides released by muscle fibers during contraction. More than 3,000 myokines have been identified, but the functions of many remain unexplored. Research indicates that myokine secretion during exercise can significantly impact metabolic health [9]. Notably, interleukin-6 (IL-6) and fractalkine are among the myokines that have garnered attention for their roles in metabolic regulation and inflammation. Recent evidence suggests that physical exercise may modulate both IL-6 and fractalkine levels, potentially impacting inflammatory processes and glucose metabolism [10].

IL-6 is a multifunctional cytokine with both pro-inflammatory and anti-inflammatory properties. Chronically elevated levels of IL-6 contribute to insulin resistance and systemic inflammation, playing a role in the pathogenesis of T2DM. However, during exercise, IL-6 is acutely released from skeletal muscle, where it acts in an autocrine manner to directly enhance insulin sensitivity and glucose uptake in the muscle itself [11]. This process activates key pathways, such as AMP-activated protein kinase (AMPK), which enhances the translocation of glucose transporter proteins (GLUT4) to muscle cell membranes, thereby improving glucose metabolism. Patients with T2DM, who have higher baseline insulin resistance, may benefit the most from the acute release of IL-6 during exercise, highlighting its potential as a powerful intervention for metabolic health [12,13]. This suggests that exercise-induced IL-6 release has a powerful role in improving metabolic health, particularly in muscle tissue, by enhancing insulin action in an autocrine fashion.

Fractalkine (CX3CL1), on the other hand, is a chemokine produced in both adipose tissue and skeletal muscle. It plays a crucial role in immune cell recruitment and adhesion, integral to the inflammatory response [14]. Fractalkine is also involved in the regulation of pancreatic islet β-cell function and has been independently associated with markers of insulin resistance, such as Homeostatic Model Assessment of Insulin Resistance (HOMA-IR) [15]. Emerging evidence suggests that exercise can modulate fractalkine levels, influencing inflammatory processes and glucose metabolism [16].

Given the significant roles of IL-6 and fractalkine in metabolic health, understanding their regulation through exercise in both healthy and diabetic individuals is imperative. Considering the close association between sedentary behavior and the onset of T2DM, exploring the causal relationships among factors secreted by skeletal muscles during both rest and contraction and the development of T2DM is crucial. Myokine secretion profiles can vary significantly between these groups, influencing their metabolic responses and overall health outcomes. By examining these differences, we can gain insights into tailored exercise interventions that maximize benefits for patients with T2DM.

This study aims to observe the effect of acute moderate-intensity exercise on circulating myokine levels, specifically IL-6 and fractalkine, in both healthy and diabetic participants. Additionally, it seeks to correlate these changes with glucose metabolism to better understand how exercise-induced myokine modulation can improve diabetes management strategies. By elucidating these mechanisms, this research aims to contribute to the development of more effective therapeutic approaches for T2DM.

## Materials and methods

Study design and approval

This prospective comparative experimental study, conducted from October 7, 2022, to December 24, 2024, was approved by the Institutional Ethical Committee and carried out in accordance with the Declaration of Helsinki.

Participants

Seventy participants were enrolled and divided into two groups: Group I, consisting of 35 individuals with T2DM, and Group II, consisting of 35 age- and gender-matched healthy individuals aged 30-60 years. It is important to note that although gender matching was not strictly enforced, a chi-square analysis (χ² = 3.534, *P* = 0.060) showed no significant gender difference between the two groups. The sample size, determined using G*Power (version 3.1.2), was based on a power of 80%, a significance level of 5%, and an effect size of 0.8, ensuring sufficient statistical power and accounting for potential dropouts.

Participants for the study were recruited from a tertiary care teaching hospital. They were thoroughly informed about the study procedure, and an information sheet was provided and explained to them. Written informed consent was obtained from those who agreed to participate, and they were then enrolled in the study.

Inclusion and exclusion criteria (for cases)

Participants were included based on the American Diabetes Association Classification 2023 guidelines. Individuals diagnosed with T2DM were included in the study if they had fasting plasma glucose (FPG) levels greater than 126 mg/dL (7.0 mmol/L) or glycated hemoglobin (HbA1c) levels above 6.5% [17]. Exclusion criteria were as follows: individuals with type 1 diabetes mellitus, alcohol use, inflammatory diseases, comorbid conditions (e.g., cancer, immunodeficiency, autoimmune diseases), current smokers, athletes, and those with diabetic complications such as foot ulcers, retinopathy, nephropathy, autonomic neuropathy, cardiovascular risks, or musculoskeletal disorders.

Inclusion and exclusion criteria (for controls)

The study included healthy, normal subjects as participants, with these attendees serving as controls alongside the cases. Exclusion criteria for controls included individuals with T2DM, type 1 diabetes mellitus, alcohol use, inflammatory diseases, comorbid conditions (e.g., cancer, immunodeficiency, autoimmune diseases), current smoking, athletes, and those with musculoskeletal disorders.

Medical evaluation and measurements

Before participating, all individuals underwent a thorough medical evaluation to identify any underlying cardiac contraindications. Their medical history was documented, and anthropometric measurements, including height (cm), weight (kg), and body mass index (BMI), were recorded. BMI was calculated by dividing weight (kg) by the square of height (m²). The resting heart rate was recorded. Systolic and diastolic blood pressures (mmHg) were measured using standardized tools and protocols.

Exercise protocol

The exercise regimen was designed by reviewing relevant literature on exercise protocols from similar studies, as no formal predefined protocol was used. The total exercise duration was 30 minutes, comprising a five-minute warm-up, followed by 20 minutes of moderate-intensity treadmill walking, and a five-minute cool-down. Moderate intensity was maintained at 64%-76% of maximum heart rate (MHR) [18]. Treadmill speed and incline were adjusted to maintain the target heart rate intensity. Participants performed the exercise under supervision to ensure adherence to the prescribed intensity. Ethical and research approval was obtained before the commencement of the study, and the exercise protocol adhered to ethical guidelines to ensure the safety and well-being of participants throughout the study.

Blood sample collection and analysis

Blood samples were drawn from the antecubital vein after a minimum of eight hours of fasting, just before (pre) and immediately after (post) exercise. HbA1c was measured using a Bio-Rad D-10 Analyzer (Bio-Rad Laboratories, Hercules, CA). FPG levels were measured using the glucose oxidase-peroxidase method. Fasting insulin levels were measured using an ELISA Kit (DiaMetra, Milano, Italy). Fasting glucose and fasting insulin levels were used to determine the homeostatic model assessment of insulin resistance (HOMA-IR) values, calculated as follows: HOMA = [(fasting insulin, mU/L) × (fasting glucose, mg/dL)]/405, which assess insulin sensitivity and, therefore, the metabolic health of the participants [19]. Blood samples were immediately centrifuged at 3000 rpm for 10 minutes to isolate serum, which was stored at -80 °C. Circulating IL-6 and fractalkine levels were assayed using commercially available ELISA kits (IL-6 ELISA Kit by R&D Systems, Minneapolis, and Fractalkine ELISA Kit by BT LAB, China), following the manufacturer's instructions.

Data analysis

Statistical analysis was conducted using SPSS version 22 (IBM Corp., Armonk, NY). Descriptive statistics, including mean ± standard deviation, were calculated for continuous variables. Paired t-tests were used to assess differences in pre- and post-exercise myokine levels within the same group, while unpaired t-tests (independent t-tests) were used to assess differences between the case and control groups. Correlations between myokine levels and glucose metabolism were evaluated using Pearson correlation coefficients. Statistical significance was set at a *P*-value of less than 0.05, with a 95% confidence level. All analyses were conducted using a two-tailed approach.

## Results

Seventy subjects included in this study were divided into two groups: Group I, consisting of 35 individuals diagnosed with T2DM, and Group II, comprising 35 healthy individuals.

Table 1 illustrates the gender distribution among cases and controls. While the study did not strictly match cases and controls by gender, a chi-square analysis indicated no significant difference in gender distribution between groups (χ² = 3.534, *P* = 0.060).

**Table 1:** Demographic characteristics: gender distribution of participants.

Gender	Cases	Percentage	Control	Percentage
Female	13	37.14%	17	48.57%
Male	22	62.86%	18	51.43%
Total	35	100%	35	100%

Table 2 summarizes the baseline parameters of diabetic and non-diabetic individuals. Significant differences were noted in weight (*P* = 0.004) and BMI (*P* = 0.001), indicating higher values among diabetic patients, while age and height showed no significant differences.

**Table 2:** Baseline parameters of groups. A *P*-value of less than 0.05 is statistically significant. S, significant; HS, highly significant; NS, non-significant; BMI, body mass index; SBP, systolic blood pressure; DBP, diastolic blood pressure; HbA1c, hemoglobin A1c

Parameter	Diabetic, mean ± SD	Non-diabetic, mean ± SD	Paired t-test	*P*-value
Age (years)	49.94 ± 5.65	47.57 ± 6.80	1.587	0.117 (NS)
Height (cm)	166.80 ± 6.53	165.17 ± 9.11	0.860	0.393 (NS)
Weight (kg)	73.09 ± 10.87	65.74 ± 9.39	3.025	0.004 (S)
BMI (kg/m^2^)	26.23 ± 3.46	23.90 ± 2.23	3.348	0.001 (HS)
SBP (mmHg)	126.63 ± 13.83	121.03 ± 9.18	1.996	0.050 (NS)
DBP (mmHg)	80.34 ± 8.32	77.66 ± 5.28	1.612	0.112 (NS)
HbA1c (%)	7.09 ± 0.7	5.22 ± 0.11	15.604	0.001 (HS)

The mean systolic blood pressure (SBP) and diastolic blood pressure (DBP) in the diabetic group were 126.63 ± 13.83 mmHg and 80.34 ± 8.32 mmHg, respectively, while in the non-diabetic group, the values were 121.03 ± 9.18 mmHg and 77.66 ± 5.28 mmHg. The differences in these values between the two groups were statistically nonsignificant. HbA1c values in the diabetic group were 7.09 ± 0.7%, while in the non-diabetic group, they were 5.22 ± 0.11%. The difference in HbA1c values between the two groups was highly significant from a statistical perspective.

Table 3 shows that, in the diabetic group, significant improvements were observed post-exercise across several key variables. Fasting blood sugar (FBS) decreased from 149.54 ± 23.29 to 133.66 ± 26.21 mg/dL (*P* = 0.001), insulin levels dropped from 14.32 ± 2.45 to 13.59 ± 2.58 µU/mL (*P* = 0.001), and HOMA-IR improved from 5.28 ± 1.19 to 4.46 ± 1.15 (*P* = 0.001). Additionally, IL-6 levels showed a substantial rise from 27.81 ± 28.87 to 195.75 ± 133.54 (*P* = 0.001), while fractalkine (CX3CL1) decreased from 4.60 ± 2.39 to 3.27 ± 2.54 (*P* = 0.001), highlighting the beneficial effects of exercise on metabolic health in diabetic individuals.

**Table 3:** Pre-post exercise biochemical levels in the diabetic group. A *P*-value of less than 0.05 is statistically significant. S, significant; HS, highly significant; NS, non-significant; FBS, fasting blood sugar; F insulin, fasting insulin; HOMA-IR, Homeostatic Model Assessment of Insulin Resistance; IL-6, interleukin-6

Variables	Pre-exercise in the diabetic group, mean ± SD	Post-exercise in the diabetic group, mean ± SD	Paired t-test	*P*-value
FBS (mg/dL)	149.54 ± 23.29	133.66 ± 26.21	11.081	0.001 (HS)
F Insulin (mIU/L)	14.32 ± 2.45	13.59 ±2.58	10.535	0.001 (HS)
HOMA (IR)	5.28 ± 1.19	4.46 ± 1.15	14.959	0.001 (HS)
IL-6 (pg/mL)	27.81 ± 28.87	195.75 ± 133.54	10.372	0.001 (HS)
Fractalkine/CX3CL1 (ng/mL)	4.60 ± 2.39	3.27 ± 2.54	9.269	0.001 (HS)

Table 4 shows that, in the non-diabetic group, exercise led to significant reductions in key metabolic markers. FBS decreased from 87.29 ± 3.82 to 82.26 ± 4.08 mg/dL (*P* = 0.001), insulin levels fell from 8.32 ± 0.35 to 7.77 ± 0.61 µU/mL (*P* = 0.001), and HOMA-IR improved from 1.79 ± 0.13 to 1.58 ± 0.18 (*P* = 0.001). Additionally, IL-6 levels increased dramatically from 20.44 ± 24.82 to 170.40 ± 69.84 (*P* = 0.001), while fractalkine (CX3CL1) decreased from 1.77 ± 0.94 to 1.31 ± 0.72 (*P* = 0.001), underscoring the positive impact of exercise on metabolic health.

**Table 4:** Pre-post exercise biochemical levels in the non-diabetic group. A *P*-value of less than 0.05 is statistically significant. S, significant; HS, highly significant; NS, non-significant; FBS, fasting blood sugar; F insulin, fasting insulin; HOMA-IR, Homeostatic Model Assessment of Insulin Resistance; IL-6, interleukin-6

Variables	Pre-exercise in the non-diabetic group, mean ± SD	Post-exercise in the non-diabetic group, mean ± SD	Paired t-test	*P*-value
FBS (mg/dL)	87.29 ± 3.82	82.26 ± 4.08	9.652	0.001 (HS)
F Insulin (mIU/L)	8.32 ± 0.35	7.77 ± 0.61	8.075	0.001 (HS)
HOMA (IR)	1.79 ± 0.13	1.58 ± 0.18	11.427	0.001 (HS)
IL-6 (pg/mL)	20.44 ± 24.82	170.40 ± 69.84	15.118	0.001 (HS)
Fractalkine/CX3CL1 (ng/ml)	1.77 ± 0.94	1.31 ± 0.72	4.092	0.001 (HS)

Table 5 illustrates significant differences in pre-exercise biochemical levels between diabetic and non-diabetic groups. Diabetic individuals had markedly higher FBS (149.54 ± 23.29 mg/dL vs. 87.29 ± 3.82 mg/dL, *P* = 0.001), insulin levels (14.32 ± 2.45 µU/mL vs. 8.32 ± 0.35 µU/mL, *P* = 0.001), and HOMA-IR (5.28 ± 1.19 vs. 1.79 ± 0.13, *P* = 0.001). Although IL-6 levels showed no significant difference (*P* = 0.256), fractalkine (CX3CL1) was significantly elevated in the diabetic group (4.60 ± 2.39 vs. 1.77 ± 0.94, *P* = 0.001), indicating critical metabolic imbalances associated with diabetes.

**Table 5:** Pre-exercise biochemical levels in diabetic and non-diabetic groups. A *P*-value of less than 0.05 is statistically significant. S, significant; HS, highly significant; NS, non-significant; FBS, fasting blood sugar; F insulin, fasting insulin; HOMA-IR, Homeostatic Model Assessment of Insulin Resistance; IL-6, interleukin-6

Variables	Diabetic group, mean ± SD	Non-diabetic group, mean ± SD	t-test	*P*-value
FBS (mg/dL)	149.54 ± 23.29	87.29 ± 3.82	15.604	0.001 (HS)
F Insulin (mIU/L)	14.32 ± 2.45	8.32 ± 0.35	14.320	0.001 (HS)
HOMA (IR)	5.28 ± 1.19	1.79 ± 0.13	17.197	0.001 (HS)
IL-6 (pg/mL)	27.81 ± 28.87	20.44 ± 24.82	1.145	0.256 (NS)
Fractalkine/CX3CL1 (ng/mL)	4.60 ± 2.39	1.77 ± 0.94	6.510	0.001 (HS)

Table 6 shows significant differences in post-exercise biochemical levels between diabetic and non-diabetic groups. Diabetic individuals continued to exhibit higher FBS (133.66 ± 26.21 mg/dL vs. 82.26 ± 4.08 mg/dL, *P* = 0.001), insulin levels (13.59 ± 2.58 µU/mL vs. 7.77 ± 0.61 µU/mL, *P* = 0.001), and HOMA-IR (4.46 ± 1.15 vs. 1.58 ± 0.18, *P* = 0.001). Although IL-6 levels did not differ significantly (*P* = 0.323), fractalkine (CX3CL1) remained significantly elevated in the diabetic group (3.27 ± 2.54 vs. 1.31 ± 0.72, *P* = 0.001), highlighting ongoing metabolic challenges even after exercise.

**Table 6:** Post-exercise biochemical levels in diabetic and non-diabetic groups. A *P*-value of less than 0.05 is statistically significant. S, significant; HS, highly significant; NS, non-significant; FBS, fasting blood sugar; F insulin, fasting insulin; HOMA-IR, Homeostatic Model Assessment of Insulin Resistance; IL-6, interleukin-6

Variables	Diabetic group, mean ± SD	Non-diabetic group, mean ± SD	t-test	*P*-value
FBS (mg/dL)	133.66 ± 26.21	82.26 ± 4.08	11.464	0.001 (HS)
F Insulin (mIU/L)	13.59 ± 2.58	7.77 ± 0.61	12.975	0.001 (HS)
HOMA-IR	4.46 ± 1.15	1.58 ± 0.18	14.709	0.001 (HS)
IL-6 (pg/mL)	195.75 ± 133.54	170.40 ± 69.84	0.995	0.323 (NS)
Fractalkine/CX3CL1 (ng/mL)	3.27 ± 2.54	1.31 ± 0.72	4.403	0.001 (HS)

Table 7 illustrates the correlation analysis in the diabetic group post-exercise, which revealed no significant relationships between IL-6 and FBS (*r* = 0.010, *P* = 0.956), insulin (*r* = 0.021, *P* = 0.906), or HOMA-IR (*r* = 0.001, *P* = 0.995). Similarly, fractalkine (CX3CL1) showed weak correlations with FBS (*r* = 0.137, *P* = 0.433), insulin (*r* = 0.081, *P* = 0.642), and HOMA-IR (*r* = 0.142, *P* = 0.417).

**Table 7:** Correlation analysis among the diabetic group (post-exercise). A *P*-value of less than 0.05 is statistically significant. FBS, fasting blood sugar; F insulin, fasting insulin; HOMA-IR, Homeostatic Model Assessment of Insulin Resistance; IL-6, interleukin-6

Pearson correlation		FBS	F insulin	HOMA-IR
IL-6 (pg/mL)	*r*-value	0.010	0.021	0.001
	*P*-value	0.956	0.906	0.995
Fractalkine/CX3CL1 (ng/mL)	*r*-value	0.137	0.081	0.142
	*P*-value	0.433	0.642	0.417

Table 8 shows that in the non-diabetic group, IL-6 also exhibited no significant correlations, while fractalkine (CX3CL1) showed slight positive correlations with fasting insulin (*r* = 0.191, *P* = 0.272) and HOMA-IR (*r* = 0.212, *P* = 0.222), indicating limited metabolic associations post-exercise in both groups.

**Table 8:** Correlation analysis among the non-diabetic group (post-exercise). A *P*-value of less than 0.05 is statistically significant. FBS, fasting blood sugar; F insulin, fasting insulin; HOMA-IR, Homeostatic Model Assessment of Insulin Resistance; IL-6, interleukin-6

Pearson correlation		FBS	F Insulin	HOMA-IR
IL-6 (pg/mL)	*r*-value	-0.004	-0.076	0.009
	*P*-value	0.982	0.664	0.958
Fractalkine/CX3CL1 (ng/mL)	*r*-value	0.152	0.191	0.212
	*P*-value	0.382	0.272	0.222

## Discussion

The role of exercise in managing T2DM is well-documented, particularly in improving insulin sensitivity and glycemic control. Recent research has increasingly highlighted the importance of myokines - biologically active cytokines released from skeletal muscle during exercise - in regulating glucose metabolism and inflammation in patients with T2DM [3,16].

This study highlights the profound impact of acute moderate-intensity exercise on myokine levels and metabolic parameters in diabetic individuals, emphasizing the potential of exercise-induced myokines as a cornerstone of diabetes management.

Our findings confirm that post-exercise measurements revealed substantial reductions in FBS, insulin levels, and HOMA-IR (Table 3), suggesting significant improvements in insulin sensitivity and glucose metabolism. These results are consistent with previous studies by Ambelu and Teferi and Colberg et al., further affirming exercise as a crucial intervention for driving favorable biochemical changes in diabetes management [20-22]. Exercise enhances insulin sensitivity, enabling muscle cells to more effectively uptake glucose, regardless of insulin availability, by utilizing glucose for energy during muscle contractions [23]. Regular physical activity enhances both insulin-dependent and independent mechanisms for skeletal muscle glucose uptake, leading to sustained improvements in insulin sensitivity and glucose disposal [24]. Additionally, Magkos et al. demonstrated in their study that acute exercise bouts can boost glucose uptake by up to fivefold through insulin-independent glucose transport mechanisms [25].

The notable increase in IL-6 post-exercise is particularly significant, and our results align with those of Garneau et al. and Sabaratnam et al. [26,27]. Traditionally viewed as a pro-inflammatory cytokine, IL-6 plays a crucial dual role in metabolic regulation, particularly during exercise. Elevated IL-6 levels enhance insulin sensitivity and promote glucose uptake in muscle tissue. Additionally, muscle-derived IL-6 has direct anti-inflammatory effects, contributing to improved glucose tolerance. Studies suggest that moderate acute increases in IL-6 can suppress tumor necrosis factor-alpha (TNF-α) production and inhibit IL-1β signaling, while IL-6 associated with physical activity stimulates the release of anti-inflammatory cytokines such as IL-1ra and IL-10 [28-30]. The significant rise in IL-6 observed in diabetic individuals suggests that exercise-induced myokine release may foster a more favorable metabolic environment, potentially mitigating some of the adverse effects of diabetes [31].

Mounting evidence indicates that proinflammatory chemokines and cytokines play a crucial role in creating conditions conducive to insulin resistance, type 2 diabetes (T2D), and related complications [32-35]. Fractalkine (CX3CL1), which acts as a chemoattractant and adhesion factor, is released from adipocytes and plays a pivotal role in recruiting inflammatory cells and facilitating their settlement at inflammation sites, such as expanding white adipose tissue in cases of obesity and T2D [14]. Post-exercise reductions in fractalkine levels highlight the anti-inflammatory benefits of physical activity, echoing the findings of Kumar et al. and Shah et al. [36-37]. The chronic elevation of fractalkine (CX3CL1) from adipose tissue is associated with inflammation and insulin resistance, suggesting that exercise may counteract the inflammatory pathways linked to metabolic dysfunction in diabetes. This reduction in the elevated fractalkine not only improves the overall metabolic profile of individuals with diabetes but also underscores the role of exercise in promoting health [9]. While previous studies have shown both increases and decreases in fractalkine levels depending on the type of exercise and the subjects, our findings demonstrate a reduction in fractalkine levels, which is associated with improved glucose disposal.

While our correlation analyses did not reveal direct relationships between myokine levels and glucose metabolism parameters, the observed changes still indicate a positive shift in the inflammatory and metabolic landscape for diabetic individuals. This disconnect may suggest that myokines, while crucial to the physiological response to exercise, interact with other factors that influence glucose metabolism in complex ways.

A strength of our study lies in its controlled approach to analyzing myokine responses and metabolic improvements following acute exercise. This focused design enables a deeper understanding of the immediate effects of exercise on myokine release and glucose metabolism in patients with T2DM, which could serve as a foundation for future research. For example, future studies could examine specific myokines released during different exercise modalities (aerobic vs. resistance training), as well as the long-term effects of consistent exercise interventions on metabolic health. 

Limitations of the study

It’s possible that the analysis focusing on a single bout of acute exercise may not capture the full picture. Acute responses can vary significantly from chronic adaptations, and myokine levels might fluctuate after different types of exercise or training regimens. Long-term studies observing repetitive exercise could provide more comprehensive insights into how myokines influence metabolic health and glucose metabolism over time. This suggests that while acute exercise may lead to immediate myokine release, understanding the longer-term effects is crucial for evaluating their significance in metabolic health.

## Conclusions

The study underscores the beneficial effects of acute moderate-intensity exercise on myokine modulation and metabolic health in individuals with diabetes. The significant reductions in FBS, insulin levels, and insulin resistance highlight the potential of exercise as a key strategy for improving diabetes management. The findings reveal that exercise enhances IL-6 levels, which assist in glucose uptake and provide anti-inflammatory benefits. Additionally, exercise reduces fractalkine levels, a chemokine linked to insulin resistance, further supporting the anti-inflammatory effects of physical activity. By promoting myokine release and reducing inflammation, exercise can significantly improve insulin sensitivity and overall metabolic health. Future research should further investigate the mechanisms by which exercise-induced myokine changes influence glucose metabolism, paving the way for tailored exercise interventions that optimize diabetes management strategies. Through this understanding, we can better harness the power of exercise in combating the challenges of diabetes, ultimately improving health outcomes and quality of life for those affected.
